# Strengthening capacity to apply health research evidence in policy making: experience from four countries

**DOI:** 10.1093/heapol/czv032

**Published:** 2015-04-21

**Authors:** Sarah Hawkes, Bhupinder K Aulakh, Nidhee Jadeja, Michelle Jimenez, Kent Buse, Iqbal Anwar, Sandhya Barge, M Oladoyin Odubanjo, Abhay Shukla, Abdul Ghaffar, Jimmy Whitworth

**Affiliations:** ^1^Institute for Global Health, University College London, 30, Guilford Street, London, WC1N 1EH, UK,; ^2^Alliance for Health Policy and Systems Research, Geneva, Switzerland,; ^3^Wellcome Trust, London, UK,; ^4^The Joint United Nations Programme on HIV/AIDS, Geneva, Switzerland,; ^5^International Center for diarrhoeal disease Research, Bangladesh, Bangladesh,; ^6^Centre for Operations Research and Training, India,; ^7^Nigerian Academy of Science, Lagos, Nigeria, and; ^8^Support for Advocacy and Training to Health Initiatives, Pune, India,

**Keywords:** Capacity strengthening, evidence-informed policy, policy

## Abstract

Increasing the use of evidence in policy making means strengthening capacity on both the supply and demand sides of evidence production. However, little experience of strengthening the capacity of policy makers in low- and middle- income countries has been published to date. We describe the experiences of five projects (in Bangladesh, Gambia, India and Nigeria), where collaborative teams of researchers and policy makers/policy influencers worked to strengthen policy maker capacity to increase the use of evidence in policy. Activities were focused on three (interlinked) levels of capacity building: individual, organizational and, occasionally, institutional. Interventions included increasing access to research/data, promoting frequent interactions between researchers and members of the policy communities, and increasing the receptivity towards research/data in policy making or policy-implementing organizations. Teams were successful in building the capacity of individuals to access, understand and use evidence/data. Strengthening organizational capacity generally involved support to infrastructure (e.g. through information technology resources) and was also deemed to be successful. There was less appetite to address the need to strengthen institutional capacity—although this was acknowledged to be fundamental to promoting sustainable use of evidence, it was also recognized as requiring resources, legitimacy and regulatory support from policy makers. Evaluation across the three spheres of capacity building was made more challenging by the lack of agreed upon evaluation frameworks. In this article, we propose a new framework for assessing the impact of capacity strengthening activities to promote the use of evidence/data in policy making. Our evaluation concluded that strengthening the capacity of individuals and organizations is an important but likely insufficient step in ensuring the use of evidence/data in policy-cycles. Sustainability of evidence-informed policy making requires strengthening institutional capacity, as well as understanding and addressing the political environment, and particularly the incentives facing policy makers that supports the use of evidence in policy cycles.

**Key Messages**

- There is widespread acknowledgement of the need to strengthen capacity to increase the use of evidence in policy cycles and that capacity needs to be developed on both the supply and demand sides of evidence production. However, little experience of capacity strengthening in health sectors in low- and middle-income countries has been published to date.
- Strengthening the capacity of individuals and organizations is necessary but probably insufficient to ensure the sustainability of evidence-informed policy making. Institutional capacity needs to be strengthened too. This requires resources, legitimacy and regulatory support from policy makers.
- Evidence of what works to develop capacity to use evidence is needed—but rarely measured. We propose a new conceptual framework to evaluate the impact of capacity strengthening activities across a variety of levels and activities.
- For sustainable change, the politics of evidence-informed policy making needs to be understood and addressed—particularly the incentives facing policy makers to support the use of evidence in policy cycles.

## Introduction

### Increasing the use of evidence in public policy decisions

Evidence-informed policy making is said to necessitate a ‘rational, rigorous and systematic approach’ (Sutcliffe and Court 2005) to the policy process which, in theory, sees evidence and analysis playing a role in decision-making. Nonetheless, the relationship between evidence and policy making is complex—not least because evidence is but one factor influencing all stages of what is in practice a ‘messy and political’ policy cycle (Buse *et al.* 2012). This relationship has been described through an abundance of theoretical and conceptual models—ranging from the relatively simplistic ‘engineering model’ (Wittrock 1991; Davis and Howden-Chapman 1996) in which good research results ‘speak for themselves’ and policy formulation follows on in an almost linear fashion, to the ‘strategic model’ which recognizes more of the political complexity involved in the willingness of policy makers to use evidence and the selective deployment of research into the politics of policy making (Weiss 1979; Hawkes *et al.* 2012).

In their review of the key components of knowledge transfer—i.e. how evidence gets incorporated into policy processes—Ward *et al.* identify five common areas, including ‘research development and selection, knowledge transfer activities and research utilization’ (Ward *et al.* 2009) while both Nutley *et al.* (2007) and Lomas *et al.* (2003) stress the importance of the characteristics of researchers (perceived as ‘credible’) or policy makers (local ownership of the research agenda, working within institutions that can access research), or both (the importance of regular interactions between researchers and policy makers) to increase the possibility of evidence influencing policy making. However, Hamel (2010) points out that while health institutions may be willing to use evidence in policy processes (including the implementation of policies), they are often ‘jeopardized by scarcity of resources to ensure that research is accessed, adapted and applied’. Additional barriers to evidence uptake in public policy (including health policy) were identified as centring on lack of access to high quality, relevant evidence and lack of a ‘timely research output’ (Oliver *et al.* 2014). Furthermore, the ‘politics of policy making’ exerts an incisive force in determining the role that evidence can play in policy making activities (Weiss 1979). 

The Canadian Health Services Research Foundation has identified several points in the policy cycle where a relatively high level of end-user (e.g. policy maker) capacity is needed for research to be incorporated into decision-making policy processes. These include: (1) the ability to acquire research evidence—either through reviewing existing literature or new commissioning to answer specific questions; (2) reviewing the strength and generalizability of evidence available; (3) adapting research findings to make them relevant in a local context; (4) evaluating the feasibility of different policy options (Lavis *et al.* 2009).

A systematic review by Clar *et al.* 2011 of the effectiveness of interventions to improve the uptake of health research evidence into policy and practice in low- and middle-income countries (LMIC) found 25 studies, 17 of which included an element of training or capacity building, 12 that fostered community participation and 1 which promoted the enhancement of health management information systems. However, of the 17 papers which included an element of training, only four specifically included building the capacity of policy makers and policy influencers to understand and use research. More detailed studies in the health field reviewing and evaluating capacity strengthening interventions alone, have been confined to single country examples—e.g. Hamel and Schrecker (2011) in Burkina Faso, or Uneke *et al.* in Nigeria—which provide useful but context-specific examples (Dobrow *et al.* 2006). Thus, there is a paucity of analysis of real-world examples of interventions aiming to strengthen the capacity of policy makers and policy-influencers to utilize research evidence.

In 2008, the Alliance for Health Systems and Policy Research at World Health Organization and the Wellcome Trust jointly issued a call for funding proposals focused on capacity development for evidence uptake in LMIC. The overall objectives of the research funding were to develop and implement innovative interventions to enhance the capacity of policy makers and/or civil society to employ health policy and systems research evidence in policy making and policy dialogue. Furthermore, teams were expected to conduct rigorous evaluations of the strategies employed. Applications were evaluated on criteria including the level of commitment to the proposal from local policy makers, local capacity development needs, level of innovation and likely impact of the intervention. Successful teams were selected from Bangladesh, Gambia, India (two teams) and Nigeria, and the programme began in 2010 with most interventions occurring in 2011–13.

A key feature of the call for proposals was that both users and providers of evidence should be involved in the bids—i.e. proposals were required to come jointly from both research groups and those in positions of policy making. This recommendation built upon the foundations established some years earlier when, for the first time, Health Ministers from 21 countries came together at a Summit (in Mexico City) to discuss health research, and specifically, the role of research in strengthening health systems and achieving the Millennium Development Goals (WHO and Government of Mexico 2004). The Summit recognized that ‘political will and good leadership’ are needed to ensure that health research is embraced within health policies, and moreover, that both the ‘push’ and ‘pull’ (supply and demand) of research utilization are key to ensuring uptake of research evidence into policy processes. Thus, Ministers at the Summit recommended fostering interactions between researchers and policy makers, and ensuring more widespread dissemination of research results so that ‘policy makers, health—care providers, the general public and funders can make better use of scientific evidence’ (WHO and Government of Mexico 2004).

A large body of literature further supports the importance of the strength of the interaction between research and policy making communities if knowledge uptake is to occur (Graham 2002). Ward *et al.*’s review of models and theories to explain how knowledge/evidence influences policy highlighted the importance of strong policy maker/researcher links as an explanatory feature in many of the models. This relationship was seen as critical to potential success in the original call, and all teams managed to successfully incorporate both research and policy making or policy-implementing communities in their bids and subsequent activities. The five research teams were based within dedicated research institutions—e.g. International center for diarrhoeal disease research, Bangladesh (ICDDR,B) Bangladesh, Centre for Operations Research and Training (CORT) India, The Centre for Innovation Against Malaria (CIAM) Gambia—or institutions supporting research as part of core functions—The Nigerian Academy of Science (NAS), and Support for Advocacy and Training to Health Initiatives (SATHI) India. A variety of health topics were tackled—ranging from reproductive health to road traffic accidents—and reflected agreed upon local health priorities.

No standardized methods of intervention or tools of evaluation were used across the five sites. Capacity strengthening activities were determined locally and based upon identified need and context. Each country team undertook their own evaluation of their activities over the 2 years, and these evaluations were then reviewed, questioned, discussed and compiled by an independent researcher (SH) who had not been involved in the original work, to identify commonalities and lessons learnt across the sites.

This remainder of this paper reports on the methods used by all five teams to build the capacity of policy makers and civil society organizations to use research evidence in policy cycles. We also report on evaluations of capacity building efforts conducted by four of the five teams and propose a new framework for evaluation.

### Pre-intervention situation analysis

In each country, a pre-intervention situation analysis was undertaken to identify needs for capacity strengthening. This included conducting interviews with key stakeholders, particularly those in positions with decision-making power, to assess needs and identify opportunities and challenges for evidence-informed policy making. Of note, however, the pre-intervention analyses did not look at the political factors indicating willingness to use evidence. The findings of the situational assessments and the proposed strategies for addressing problems identified are shown in Table 1.

**Table 1:** Results of situational assessment and strategies to address gaps

Key findings in assessment	Countr(ies)y where finding applies	Interventions to address gap
Researchers pursue their own interests	Bangladesh	Increase opportunities for researchers and policy makers to meet and share ideas
Poor communication skills of researchers and research outputs not relevant	Bangladesh	Change methods of communication—use of multimedia communications
Lack of centralized site for accessing information	Bangladesh, Gambia, Nigeria	Build infrastructural support for policy makers to access information; established shared hosted website for ease of research output access
Few opportunities for researchers and policy makers to meet	Bangladesh, Nigeria	Establish regular meetings between researchers and policy makers
Low level of political will to use evidence in policy making	Nigeria	Workshops with policy makers to emphasize need for evidence-informed policy making
Poor capacity to interpret and use data	Bangladesh, India (x2)	Training programmes for policy makers and policy influencers

In Bangladesh, a documentary review and 20 in-depth interviews were undertaken using standardized survey instruments. Interviews with policy makers highlighted some of the barriers to the use of evidence in policy processes including both supply and demand-side barriers. On the supply side, policy makers were concerned that research is not always needs-focused—i.e. researchers were more keen to pursue their own research interests rather than address the needs of the health system. Most commonly, policy makers identified the engagement/communication strategies of researchers as an important barrier to evidence uptake. Research outputs tend to be reports, dissemination seminars and peer-reviewed publications, few of which were seen as relevant to the needs of the policy making community. In addition, the content of research communications was seen as problematic. According to one senior policy making official: ‘I feel shy to go to research dissemination programmes because I do not understand their findings especially the statistical part’.

Interviews among the research community in Bangladesh identified a number of problems in promoting evidence uptake. These included: a lack of incentives to participate in policy-relevant research, particularly when compared with a large number of incentives to publish in peer-reviewed journals; and a lack of awareness of the policy making process in the country, and thus, the role that evidence could (or could not) play in policy.

The lack of any centralized site for sharing of information, and the dearth of opportunities to meet with policy makers and understand their needs and demand for evidence were seen as barriers to evidence uptake in a number of countries. The situational analysis conducted in Nigeria noted a report from the Ministry of Health highlighting a low level of political will to incorporate research evidence into policy formulation (Federal Ministry of Health (FMOH)/Policy Project Nigeria 2002), and this was compounded by the fact that <10% of Nigerian health policy makers use standard tools for evidence-informed policy making such as the Cochrane Reviews (Aluwon 2006). Moreover, a shortage of evidence-informed policy implementation guidelines and tools was found. Developing a proposal to overcome these gaps required sustained collaboration and co-operation between the research team at the Nigerian Academy of Science, and both Federal and State Ministries who are responsible not only for policy formulation but also for policy implementation.

Researchers in the Gambia completed a situational analysis with Parliamentarians on the use of evidence in health policy formulation. Based on the needs identified by Parliamentarians themselves, a number of initiatives were proposed which focused on enhancing capacity to gather and review information. Infrastru-ctural improvement (upgrading computer and internet access) along with small group training workshops formed the core of most activities.

A baseline survey by SATHI, Maharashtra, India, and conducted among health managers at district level and below, found low capacity to use and interpret data for planning. Moreover, there was a noted lack of a link between use of data and systems of accountability (including elements of reward). Thus, the SATHI team proposed to build capacity, through workshops and training programmes, among both health officials as well as local communities to use, analyse and interpret data for health planning. The involvement of community stakeholders was in line with the Indian Government’s 2007 policy of community-based monitoring in health systems. This policy involves promoting the participation of community members, service beneficiaries and non-Governmental organizations as well as service providers and health officials in community monitoring of health service data (Ministry of Health and Family Welfare 2006).

In Gujarat, India, CORT focused on the use of health information data at district level for programme planning. A baseline survey in six poorly performing districts revealed that among senior programme managers, more than one-third were classified as making ‘poor’ or ‘very poor’ use of existing datasets in their decision-making processes, with less than a third using data at a level classed as ‘excellent’ or ‘good’. Only one in ten of the decision-making officials had received any previous training in the use of health-related data. Reasons for poor use of data included concerns about the quality of the data available to them, ‘too much information’ and ‘lack of skills for data analysis’.

### Implementing the programme in each country

Interventions to enhance the capacity of policy makers (and policy influencers) varied from site to site—see Tables 1 and 2. In reviewing the activities across the five sites, we decided to use the United Nations Development Programme (UNDP) definition of capacity as “the ability of ‘individuals, institutions and societies’ to perform functions, solve problems, and set and achieve objectives in a sustainable manner” (United Nations Development Programme 2010). From this definition, capacity can be enhanced at the individual, organizational and institutional levels (Department for International Development 2010)—and we used this framework to categorize the different activities underway across the sites as we felt that this would give structure to the analysis.

**Table 2:** Methods used to enhance capacity for increasing use of evidence in policy cycles

	Enhancing individual capacity	Enhancing organizational capacity	Enhancing institutional capacity
Bangladesh, ICDDRB	Three-day workshops for policy makers, programme managers and practitioners: how to conduct literature reviews, how to evaluate evidence, how to write policy briefs.	Regular seminars between researchers and policy makers. Improved digital communications by email and text messaging.	RPCC established within government institution. Multimedia activities including website hosting.
Gambia, CIAM	Three-day workshop for Parliamentarians, plus training programmes for health journalists	Strengthening of infrastructural capacity (information technology hardware, internet routes, etc). Established web-based repository of information.	
India, CORT	Training programme for different cadres of health staff; topics included: sources of data, indicators, communication skills and use of evidence in policy making. Follow-up visits with trainees undertaken.	Incorporation of evidence-policy topics into training modules of post-graduate health-training institutes.	
India, SATHI	Three-day training courses (four over the course of a year) for local planning and monitoring committees. Content focused on health rights, health inequalities, use of data.		
Nigeria, NAS	Training workshops for health care managers, focused on health policy analysis, health systems and governance, advocacy, health economics and evidence in policy making	Biannual policy retreats with researchers, policy makers, managers.	Lagos State Ministry of Health established a Health Policy-Research Committee with commissioning, review and advisory functions.

Individual capacity building is often centred around training and knowledge transfer for individuals in a system. Building capacity at the organizational level refers to strengthening systems to enable organizations to operate effectively and efficiently. Capacity development at the institutional level means a focus on the norms and rules which govern decision-making: for example rules dictating the use of evidence, and even specific kinds of evidence, in decision-making. It can also encompass the norms governing whether or not policy makers are incentivized or sanctioned in relation to formulating policy in specific ways. Table 2 summarizes the activities in each country and categorizes them according to whether they were directed at strengthening capacity at the individual, organizational and/or institutional levels.

### Strategies for enhancing individual capacity

In all five settings, activities focused on enhancing the capacity of individual decision-makers—predominantly through training programmes and/or workshops.

The intervention in Bangladesh included an ‘executive training programme’ for policy makers, programme managers and practioners to acquire, assess, adapt, and apply evidence to improve policy and practice. The activities included a series of 3 day workshops with a focus on ‘how to apply research evidence in health policy making’ and covered topics such as how to conduct a literature search, methods for quantitative and qualitative analysis, how to judge the rigour and strength of research evidence, and how to write policy briefs. These workshops were originally intended to reach 50–60 mid-level professionals, but interest in the courses was high and eventually more than 250 were trained. The training modules have been made available online and are open access [research policy communication cell (RPCC) website].

The Nigerian team conducted two training workshops for senior- and middle-level health care managers in the Lagos state Ministry of Health on the use of research evidence to influence policy making. These workshops also aimed to increase the demand for new research in the future by encouraging links and partnerships between researchers and policy makers. The content of the training module covered a variety of public health topics including: health policy analysis; health systems governance; health economics and advocacy in health research. The published training booklet was subsequently distributed to several relevant institutions nationwide.

The programme in the Gambia aimed to increase the capacity of Parliamentarians to access and use research evidence. Three-day modular training courses were offered to all Parliamentarians—with 85% participating in the first round and 66% coming for a second enhanced round of training. The research team also undertook training programmes for journalists who report on health issues with the aim of increasing advocacy and demand for the use of evidence in health policy making processes.

In Maharashtra, the SATHI training sessions focused on questions of content (i.e. training to understand the evidence and datasets available), and also the process of health planning itself—including issues of locally appropriate resource allocation for future health plans. These training sessions were attended by members of local monitoring and planning committees who, within the framework of the Indian Government’s National Rural Health Mission (NRHM), are charged with using evidence for decentralized health planning. Four 3-day training courses were held over the course of 1 year, and participants were expected to attend all four courses. Topics were wide-ranging and included health rights, health inequalities and a more detailed focus on use of data for decentralized health planning. A similar training programme in Gujarat (the project led by CORT) was tailored to the needs of individual staff at different levels of the health system, and was undertaken by staff from a specialized post-graduate teaching institute. Topics included the use of evidence in policy decisions, data sources, key indicators and their interpretation, and effective communication skills. Training workshops were followed with repeat contacts with stakeholders.

### Strengthening organizational and institutional capacity

A smaller number of interventions focused on developing organizational capacity. The team in the Gambia supported strengthening the information technology capacity of the National Assembly through provision of desk-top computers and upgraded internet access. The team established a web-based repository of locally generated research evidence (from local studies—both peer-reviewed and ‘grey’ literature) on topics locally identified as high priority.

Other activities to enhance organizational capacity included establishing opportunities for regular interaction between researchers and policy makers. In Bangladesh, this took the form of seminars and a policy dialogue, while in Nigeria, there were biannual policy retreats where researchers, health managers and policy makers were given an opportunity to interact and discuss research findings in order to identify priority areas for health systems strengthening.

Institutional capacity development is more challenging to achieve, but the first steps to institutionalize the uptake and use of evidence in policy making across the five projects were made in Bangladesh through the establishment of a RPCC. The RPCC was set up within the government to act as a platform for providing synthesized information on reproductive health issues to policy makers. This was complemented by multimedia activities which included establishing a mobile and email network to disseminate evidence directly to policy makers, and hosting a website within the Government’s own web portal which served as a forum for sharing policy briefs and video clips from a policy discussion meeting.

#### Methods for evaluation

The evaluation of capacity strengthening activities took place in 2013 in four of the five sites (not including the site in Gujarat, India, as unforeseen delays in implementation of activities meant that there was no time for evaluation within the overall funded time-frame). Each implementing partner was responsible for conducting its own evaluation, and methods used included conducting in-depth interviews with key stakeholder to capture their understanding of process and impact (Bangladesh, Nigeria), quantitative surveys of changes in knowledge, attitudes and practice among participants in training workshops (India, SATHI, Nigeria) when compared with pre-training levels, and for one country (Gambia) a documentary analysis of the number of times that evidence was referred to in Parliamentary discussions of health issues pre- and post-intervention. These evaluations were then reviewed, discussed, analysed and combined into a single report by an independent evaluator (SH). The combined evaluation exercise looked to identify common and/or contrasting features across all sites, and to situate the interventions and evaluations within theoretical frameworks.

### Evaluation of capacity development activities

#### Individual capacity

In all four sites, a positive change was recorded in knowledge and understanding of the use of evidence. Policy makers and members of civil society organizations reported higher levels of factual knowledge concerning the appropriate use of evidence, and for some this knowledge was translated into action. In Nigeria, for example, one senior official from the State level Ministry of Health commented:

“In planning activities in my unit, we now search [the] literature. We don’t just plan activities; we ask questions; we also use past results for planning future programmes”.

A similar comment was recorded from policy making participants in the training in Bangladesh:

“Now I look for latest evidence related to my work through PubMed search and use in my practice”.

However, a note of caution was sounded by other Bangladeshi policy makers. Some were concerned that those who had participated in the training were not influential figures in policy making processes—either through lack of seniority or lack of political access. Nonetheless, these respondents also noted that any future change of government may see them move more directly in to policy-making circles, and the training would be useful at that time.

Pre- and post-intervention tests in Maharashtra found that knowledge about health planning increased, as did awareness of rights in relation to health and health services. The increase in knowledge was higher among participants from the community-based sector than among government health officers.

#### Organizational capacity

In both Bangladesh and Nigeria participants noted improvement in researcher-policy maker relationships during and after the intervention. Face-to-face communications supplemented by the establishment of dedicated policy-relevant research summary websites were seen as important methods for improving shared understanding.

Evaluation of the impact of involving community members in health planning processes in rural India noted a number of changes to plans and health service delivery. Participation of civil society representatives was judged as improving accountability for local health expenditures—and a perceived improvement in local health service function:

“An NGO [civil society organization] in our area keeps a close watch on the Primary Health Centre. They know what patients want … . They also ask for expenditure accounts for inspection … . There is drinking water and food for patients, the bed-sheets are clean because of the NGO's close watch.” (Medical Officer, Maharashtra).

In one District alone, post-intervention evaluation found that between 21 and 59% of funds were now being used to address issues identified as a priority by communities themselves.

#### Institutional capacity

Although few activities were focused in this area, the establishment of a RPCC in the Ministry of Health in Bangladesh was perceived as particularly influential. This acted not only as a ‘go to’ hub for up-to-date evidence in particular health thematic areas, but was also valued as an opportunity for increasing interactions between researchers and policy makers at a more personal level. At the end of project-based funding, the Ministry of Health and an external donor committed funds to the further activities of the RPCC.

A recommendation of the programme in Nigeria was adopted by the Lagos State Ministry of Health which set up a Health Policy-Research Committee. This multi-stakeholder committee aims to facilitate the direct assimilation of research into policy and serves to both commission and review research evidence and advise the Ministry on the implications for policy making.

## Discussion

There are substantial gaps in our general understanding of the mechanisms by which the influence of (research) evidence on policy processes, and on policy makers, can be enhanced. Much effort is directed at using empirical evidence to persuade policy makers of the superiority of one policy option over another or raising the profile of an issue on a policy agenda (Shiffman *et al.* 2002). However, fewer resources are directed at capacity development to promote greater use of research evidence among policy communities. Our description and evaluation of capacity strengthening among policy makers in four countries represents one of the few multi-country experiences and provides valuable lessons for others concerned with the uptake and utilization of evidence in health policy.

This article has described five programmes of implementing activities for a shared goal, that of enhancing capacity to increase the uptake of evidence in policy cycles. The context and setting for each programme varied greatly. The central role of the political context surrounding decisions concerning research uptake and research utilization has been recognized for decades (Weiss 1979; Hanney *et al.* 2003). Despite this, one feature that was missing from each pre-intervention situational assessment was a detailed description of the way that ‘political aims and desires contribute to policy making’ (Hallsworth *et al.* 2011). It may be that the political climate and political ‘appetite’ for using evidence in each setting was in fact the greatest driver of capacity strengthening success. Unfortunately, this was not captured within the pre- or post-intervention evaluations. Nonetheless, we believe that despite the reality that politics is an inherent feature in all policy settings; there are nonetheless important lessons for future programmes of capacity strengthening to draw from our multi-country experience.

Within the definition of capacity development identified by UNDP and others, many of the activities that the five groups undertook fell under the heading of ‘individual level capacity strengthening’. Training programmes and workshops were implemented in all five sites, with a major focus on strengthening the capacity of individual policy makers to access, review and interpret evidence. The training programmes were well-received and well attended in many sites and moved beyond their original remit to include discussions on concepts such as health equity and health inequalities.

Strengthening of organizational capacity was also recognized as an important activity by several of the teams, and mechanisms and structures were established to both increase capacity to access research evidence (e.g. through data repositories, upgrading of institutional infrastructure or multimedia messaging to increase evidence coverage), as well as establishing systems to provide synthesized evidence through production of policy briefs and research summaries. Moreover, recognizing that increased interaction between policy makers and researchers is vital to increasing the uptake of evidence (UNDP, Fast Facts), several of the sites established forums to increase exchanges between these two groups—and this was particularly successful in Bangladesh.

None of the five teams undertook activities which truly fall into the domain of enhancing institutional capacity—which might include, for example, strengthening regulatory systems, ensuring equity in public service delivery, or enhancing systems of governance and accountability. Such activities are likely to be vital to ensure sustainable long-term change in the culture of using evidence in policy cycles, but they are ambitious and long-term activities usually beyond the capacities of individual specialist research teams such as represented in this programme.

It is possible, however, that developing individual and organizational capacity is a pre-requisite for seeing long-term institutional change. Previous examples (not from this programme) include the Health Policy Advisory Committee (HPAC) in Nigeria’s Ebonyi State which was established to ‘bridge the gap between researchers and policy makers’, with activities including analysis and sharing of information relevant to health policy decisions (Uneke *et al.* 2012). Lagos State (Nigeria) has now commenced the process of setting up a similar body, the Health Policy-Research Committee. In Kenya, the Wellcome Trust-supported Consortium for National Health Research (CNHR) provides ‘targeted support … to policy makers charged with regulating and co-ordinating health research activities’. Both the HPAC and CNHR focus on strengthening the capacity of individual researchers, and promote organizational capacity by encouraging increased interaction between policy makers, researchers and others involved in decision-making. Following a similar model within this funding scheme, the team in Bangladesh through the establishment of the RPCC to act as a platform for sharing of evidence and discussion of policy options between researchers and policy makers. The RPCC was supplemented by an extensive programme of individual level capacity building.

The RPCC (this programme), HPAC and CNHR (previous examples from elsewhere), represent attempts to institutionalize the use of evidence, but are not backed up by regulatory frameworks which necessitate the use of evidence in policy making. This is the domain of developing institutional capacity and it requires government support and ongoing resource commitments and incentives. For example, the United Kingdom’s National Institute for Health and Care Excellence (NICE) is a public body providing evidence-based guidance for health and public health practitioners, through ‘a rigorous process that is centred on using the best available evidence and includes the views of experts, patients and carers, and industry’ (NICE website). The NICE guidelines, which focus predominantly on questions of efficacy and cost-effectiveness, are mandatory within the UK’s national health system.

In the absence of institutional norms and regulations around the use of evidence, there may be a concern that decisions around health policy are subject more to value-driven decision-making than evidence-informed processes (Clark and Weale 2012) or even more parochial norms and interests of decision makers (Buse *et al.* 2012). However, capacity for the access, analysis and interpretation of evidence may lie outside the remit of policy makers, and more within specialized agencies.

Evaluation of the capacity-strengthening activities across the five projects has highlighted a number of lessons learnt, which are, we believe, of interest to all those interested in seeing health policy making become more evidence-informed. First, the goal of enhancing capacity of individuals to access, understand and use evidence was a success in all four projects where evaluations took place. Pre- and post-test surveys of knowledge (both substantive and conceptual) showed improvements in test scores following interventions involving a variety of pedagogical techniques including training programmes and workshops. The project in rural Maharashtra achieved most success in building the capacity of members of civil society organizations—indicating that knowledge capacity can be strengthened not just for policy makers but also for those interested in policy influencing activities too.

The role of individuals in promoting the uptake of evidence into policy processes has been widely noted, and characterized by Banks (2008) as ‘good evidence needs good people’—which can, of course, include ‘good leaders’ calling for the use of evidence, and ‘good researchers’ promoting evidence translation and uptake. For LMIC in particular, the lack of capacity among individuals to understand and use evidence has been particularly damaging to overall goals of promoting evidence-informed policy (Gonzalez-Block and Mill 2003). Moreover, measuring the impact of capacity building goals at the level of individuals is often methodologically more feasible than building and measuring either organizational or institutional capacity.

The second lesson was that although teams in Bangladesh, Gambia, India and Nigeria implemented some aspects of building organizational and even institutional capacity (in the case of Bangladesh), the overall impact and sustainability of these inputs was not clearly measured in most countries. The teams were able to measure variables such as improvements in numbers of interactions between policy makers and researchers, or website hits on evidence/policy portals developed (e.g. in Gambia and Bangladesh), but the overall impact on policy and practice was intangible in most settings. Given that the time-frame was relatively short (2 or 3 years), and policy and planning cycles are generally longer (Hallsworth *et al.* 2011), the capacity building interventions may not yet have had an opportunity to demonstrate impact.

We found that no one single approach demonstrated a higher degree of effectiveness in strengthening capacity compared with the experience of other countries. This result is not unexpected. A review by Moore *et al.* (2011) of interventions to increase the uptake of research in population health policies noted three main strategies commonly used: increasing access to research, promoting frequent interaction and increasing organizational research receptivity. The findings of the review indicate that no one method/approach that was effective in all settings, but the studies did tend to show a tendency towards greater use of evidence if policy makers had increased access to timely and relevant research. We used Moore’s framework of three strategies to categorize the capacity strengthening activities. We found that the five programmes described in this article used each of Moore’s three main strategies (access, interaction and receptivity), but like Moore, we were not able to identify one method as being more effective than any other. We believe that this illustrates the highly context-specific nature of capacity strengthening programmes, but may also be a reflection of the underlying willingness/motivation of policy makers to use evidence in policy decisions—a parameter possibly driven more by politics than guidelines in most settings.

Finally, we have identified a lack of standardized agreement on what ‘success’ might look like within these interventions, which leads us to our final conclusion—the need for the development of outcome and impact measures to assess the overall impact of capacity strengthening interventions. Evaluating whether one approach is more successful than another is neither feasible nor perhaps desirable given the context-specific nature of policy processes, and the variety of other influences on policy cycles—including the political, economic and cultural nature of policy processes. Nonetheless, evidence of what works to develop capacity to use evidence is needed—but rarely measured.

Evaluating impact requires identifying appropriate indicators of success. In Table 3, we propose a framework for measuring and evaluating capacity strengthening activities (access, interaction, receptivity) across the three spheres (individual, organizational, institutional) as part of a more systematic approach. Thus, for example, increasing access to research evidence for individual policy makers/policy-influencers could be measured through their individual levels of access to analysed evidence—e.g. their ability to access and use systematic reviews, or other high quality evidence. In comparison, research receptivity at the institutional level could be measured through the existence of norms and policies requiring the use of evidence in policy-level decisions. These proposed indicators may help measure the effectiveness of specific capacity strengthening activities; they are not, however, designed to measure the overall impact of such activities on policy processes.

**Table 3:** Framework for measuring the impact of capacity strengthening efforts in health policy making

	Developing individual capacity	Developing organizational capacity	Developing institutional capacity
Increasing access to research evidence	Analysed research available, accessible and usable by policy makers/influencers.	Development of multimedia communications for research dissemination.	Improved infrastructural support for policy makers to access research evidence including summaries. Policy maker required to review evidence base during policy cycle—either directly, or through mandated external body.
Increasing and deepening interaction	Evidence of interaction between policy community and research community (e.g. joint meetings, workshops, etc).	Opportunities for researcher-policy maker interaction. Identification of knowledge brokers. Involvement of policy community in setting research agenda. Involvement of researchers in policy formulation	Set mechanism for consultation between researchers and policy makers at all stages of policy cycle.
Increasing research receptivity	Rates of participation in training programme. Ability to assess and critically analyse evidence.	Ongoing training programmes/opportunities established.	Norms and policies indicating requirement to use evidence in policy process decisions. Systems of accountability, including through parliamentary review, established to ensure that policy decisions are evidence-informed where appropriate.

## Conclusion

A number of lessons can be drawn from the process of implementing and evaluating programmes to strengthen the capacity of health policy makers to use research evidence. First, as evidenced by the large number of initial applications, this funding scheme has highlighted an appetite for capacity strengthening to increase use of evidence in health policies that exists among both research and policy-making communities. This is important given the mismatch between evidence and policy inherent in much of global public health (Buse and Hawkes 2013). Each of the five successful teams of applicants represented a partnership between academic researchers and policy makers or programme implementers. Many of the funded activities were innovative and ground-breaking attempts to increase the use of evidence in policy making decisions, perhaps as a result of these partnerships.

Second, while the term ‘capacity strengthening’ may be widely understood, there were significant variations in what constitutes ‘evidence’ in health policy. Although researchers may promote ‘high quality’ evidence such as that from systematic reviews or meta-analyses, communities and service users may have a different opinion of the evidence that ‘counts’. In at least three of the sites, there was a focus on increasing capacity to use routine health management data—sometimes in conjunction with research evidence, and sometimes alone. The importance of routine data collection is well understood in many settings, particularly within the context of disease-specific targets and goals for health system performance—which is what service users actually experience (Boerma *et al.* 2010). However, as projects such as Data for Decision-Making have shown, the capacity to review, analyse and utilize the data for public health decisions is often lacking (Azubuike and Ehiri 1999; Pappaioanou *et al.* 2003). Although the need to increase the analysis and use of such data is well recognized (Lavis *et al.* 2009) there are few existing examples of building the capacity of end-users to analyse and use these datasets in health planning (Pappaioanou *et al.* 2003).

Third, evidence plays a role at all levels of the policy cycle and the health system. From agenda setting through to policy formulation, evidence can be used by a variety of actors employed at different levels of the system. The five teams undertook capacity strengthening with a variety of stakeholders ranging from local representatives of civil society and sub-district health officers, through to Parliamentarians. Building capacity for using and applying evidence was seen to be important across the stakeholder spectrum.

Fourth, the political nature of the use of evidence needs to be more widely acknowledged. We have seen that policy makers can be ‘educated’ on the benefits of evidence-informed policy, they can be provided with tools to access, analyse and utilize evidence, and can be encouraged to engage more closely with researchers. We can even promote more reflexive policy processes from policy makers, but we should not lose sight of a fundamental issue—policy is not simply about soft persuasion but also hard bargaining. Policy change, including evidence-informed health policy change, is inherently political. Political in the sense that change will redistribute authority and/or resources. In the five settings described in this article, such potential redistribution included the monitoring of accountability from health systems to communities (in India), and decisions around research resource allocation in Bangladesh and Nigeria. Decision makers, whatever their station in the policy process, will be aware of the ‘politics’ of change. These politics will condition, to differing degrees, depending on the extent of the interests at stake, both the ability (capacity) and willingness of decision makers to take evidence into account. In short, it is important to be aware that policy challenges are not solely technical questions in search of systematic application of specialist expertise. A failure by the evidence-to-policy advocacy movement to appreciate that there are winners and losers, or even that political will and leadership is needed to cajole others to support change, can only result in frustration. All else being equal, we need to identify the incentives for policy makers to act on evidence.

This leads us to our final conclusion which addresses the long-term sustainability of capacity-strengthening efforts. Although identifying successful methods for enhancing individual and organizational capacity may be a vital first step for seeing improvements in the use of evidence, sustainable changes can only happen through developing the capacity of the institutions that can provide the incentives for individuals and organization to adopt more evidence-informed decision making. In other words, we are advocating for an institutionalized rather than an ad hoc approach to enhancing capacity to apply evidence. This could be achieved through the establishment of an external body (such as NICE or RPCC) since this removes the need for strengthening the capacity of each and every policy maker who comes into post. However, such an approach requires resources, legitimacy and regulatory support from policy makers themselves—in other words, it requires political support. Strengthening the appreciation and capacity of individual policy makers and their organizations to make greater use of evidence is a necessary first step in generating better evidence informed policy. Building sustainable institutional capacity will be a more challenging but vital further step. Recognizing that politics is an inherent element of policy making will condition both of these steps, but does not invalidate them. Ultimately, capacity for evidence-informed policy will be a significant determinant of our collective ability to bring about health for all.
